# Assessment of sleep problems with the Insomnia Severity Index (ISI) and the sleep item of the Patient Health Questionnaire (PHQ-9) in cancer patients

**DOI:** 10.1007/s00520-021-06282-x

**Published:** 2021-05-29

**Authors:** Thomas Schulte, Dirk Hofmeister, Anja Mehnert-Theuerkauf, Tim Hartung, Andreas Hinz

**Affiliations:** 1 Rehabilitation Clinic Bad Oexen, Bad Oeynhausen, Germany; 2 Department of Medical Psychology and Medical Sociology, University of Leipzig, 04103 Leipzig, Germany; 3 Jewish Hospital, Berlin, Germany

**Keywords:** Cancer, Insomnia, Oncology, Psychometrics, Psycho-oncology, Sleep disturbances

## Abstract

**Objective:**

The objectives of this study were to examine sleep problems in cancer patients, to test the psychometric properties of the Insomnia Sleep Index (ISI) in comparison with the sleep item of the Patient Health Questionnaire-9 (PHQ-9), and to analyze disrupting factors which might cause the sleep problems.

**Methods:**

A sample of 1026 mixed-site cancer patients in treatment at a German oncological rehabilitation clinic was examined.

**Results:**

The reliability of the ISI was very good (Cronbach’s alpha = 0.92), and the results of the confirmatory factor analysis were acceptable. Females reported worse sleep quality (ISI mean: 13.7 ± 6.6) than males (10.7 ± 6.4). Sleep problems as measured with the PHQ-9 sleep item were markedly higher than those in the general population (effect size *d* = 1.15).

Patients reported that, of the factors that disrupted their sleep, psychological factors (brooding, worries) were more relevant than symptom factors (pain, nocturnal urination, or restless legs).

**Conclusions:**

The ISI is effective in detecting sleep problems in cancer patients. Normative studies with the ISI would be helpful for assessing ISI mean scores. Sex differences should be taken into account when groups of patients are compared. The sleep item of the PHQ-9 can be used in epidemiological studies.

## Introduction

Sleep problems are frequent in cancer patients [1]; the prevalence of sleep disturbances has been found to range from 30 to 93% [2]. Sleep problems often do not disappear after cancer treatment [3], and they are associated with reduced quality of life [4, 5], depression [6], impaired concentration [7, 8], and even reduced survival rates [9, 10]. In clinical practice, sleep problems often remain undetected and untreated despite the high prevalence [11, 12].

Several questionnaires have been developed for effectively measuring sleep quality [13]. One of these instruments is the 7-item Insomnia Severity Index (ISI) [14]. Its development was based on the diagnostic criteria for insomnia outlined in the fourth edition of the Diagnostic and Statistical Manual of Mental Disorders (DSM-IV) and the International Classification of Sleep Disorders (ICSD). Reliability estimates of the ISI in terms of Cronbach’s alpha range from 0.74 to 0.92 [15]. While the ISI is generally used as a one-dimensional scale, several studies tested the factorial structure and obtained mixed results, with two-factorial [15, 16] and three-factorial [17, 18] solutions.

Age and sex differences in sleep quality have been examined by multiple studies conducted with both patient and general population samples. Many found that females report higher levels of sleep problems than males do, while no consistent age effects were observed [19–21]. Since cancer types and sex can be confounded, it is important to quantify sex differences when the impact of specific cancer types on sleep quality is to be examined. Therefore, one aim of this study was to also test age and sex differences.

In addition to questionnaires that only measure sleep quality, there are several for measuring quality of life (QoL), fatigue, or depression that include a sleep item. Examples of such instruments are: the Patient Health Questionnaire-9 (PHQ-9) [22], the General Health Questionnaire-12 (GHQ-12) [23], and the European-Organization-For-Research-And-Treatment-Of-Cancer QoL questionnaire EORTC QLQ-C30 [24]. While it is presumable that the 7-item ISI is more effective in detecting sleep problems than are the single sleep items included in questionnaires such as the PHQ-9, this supposition must be empirically tested to establish whether or not that is in fact the case. For that reason, we compared the correlations of the ISI and the PHQ-9 sleep item with several other mental health and quality of life scales.

Oncologists often pay too little attention to sleep problems when treating cancer patients [25]. Moreover, even if they do use a suitable instrument for screening sleep quality and detect poor results, those findings do not reveal why the sleep disturbances occur. Therefore, we also investigated physical, mental, and environmental disruptive factors that patients report as causing them trouble sleeping.

In summary, the aims of this study were (a) to test psychometric properties of the ISI in comparison with the sleep item of the PHQ-9 in a large sample of cancer patients; (b) to analyze the effects of sex, age, and tumor type on sleep quality; and (c) to explore the role of disruptive factors which cause the sleep problems.

## Methods

### Sample of patients

This study was performed in a German oncologic rehabilitation clinic. In Germany, most cancer patients are offered the opportunity to spend some time, often 3 weeks, at a rehabilitation clinic to help restore their physical and social functioning after cancer treatment. Inclusion criteria for this study were age 18 years or older, the absence of cognitive impairment, and sufficient command of the German language. A total of 1350 patients were asked to take part in the study. Most of the patients were informed about the background and the objectives of the study in personal interviews at the beginning of the stay in the rehabilitation clinic. Of 1350 patients who were asked to participate, 1053 (78%) were willing to do so. In most cases, the questionnaire was filled in several days after the beginning of the stay in the clinic. All study participants gave their consent to taking part in the study after having been informed of the data collection and data storage policy. The Ethics Committee of the Medical Faculty of the University of Leipzig approved the study.

### Instruments

The ISI is a seven-item questionnaire for assessing sleep problems. The items cover (1) sleep onset, (2) sleep maintenance, (3) early morning awakening, (4) satisfaction level with current sleep pattern, (5) interference with daily living, (6) noticeability of impairment due to the sleep difficulty, and (7) level of distress caused by the sleep problem. Each item is rated on a 5-point Likert scale ranging from 0 to 4, and is summed with the others to result in total scores ranging from 0 to 28. The scores can be divided into categories as follows: no significant insomnia (0–7), subthreshold insomnia (8–14), moderate insomnia (15–21), and severe insomnia (22–28) [14, 26]. In this study, we used the German translation of the ISI [27].

The PHQ-9 is a screening instrument developed to measure depression [22]. For each of the nine items, the patients are asked to assess how much they were bothered by the given symptoms over the last 2 weeks. There are four answer options: not at all (0), several days (1), more than half of the days (2), and nearly every day (3). The sum score (range 0 to 27) indicates the degree of depression. One of the items pertains to sleep: “How often have you been bothered by trouble falling or staying asleep, or sleeping too much?” Normative values of the PHQ-9 are available [28]. The PHQ-2 is a shortened form of the PHQ-9 [29].

In addition to the ISI and the PHQ-9, we used the following questionnaires: the Generalized Anxiety Disorder screener GAD-2, measuring anxiety with two items [30, 31], the Adjustment Disorder New Module-8 (ADNM-8), a brief version of the ICD-11 adjustment disorder scale [32, 33], the Work Ability Score (WAS), a single-item measure for assessing work ability on a 0–10 scale [34], and an adapted version of the Diagnostic Criteria (DC) for measuring cancer-related fatigue. Here, we used the 11 criteria for measuring cancer-related fatigue developed by the Fatigue Coalition [35] and adopted a four-point Likert scale for each item [36]. Finally, we assessed QoL with the two-item general health/QoL scale of the EORTC QLQ-C30 [24].

To explore physical and mental factors that might have precipitated their sleep problems, the patients had to respond to 12 items representing potential causes. The factors were pain, nocturnal urination, brooding, worries, unsolved problems, sweat, hot flashes, cold feeling, restless legs, breathing difficulties, nightmares, and noise. The answer options for each of the factors ranged from 0 (not at all) to 3 (strongly). We calculated the mean scores of the answers and the frequencies of respondents who answered with 1 (“a bit”) or greater.

### Statistical analysis

The impact of sex and age on sleep quality was tested with two-factorial analyses of variance (ANOVAs), with the age categorized into five groups as shown in Table 1. The impact of tumor category and time since diagnosis on sleep quality was tested with two-way ANOVAs including the covariate age group, separately for both sexes. Effect sized *d* were calculated according to Cohen [37]. Cronbach’s alpha coefficient was used to determine the reliability of the ISI. Linear associations between the sleep variables and other variables were calculated with Pearson correlation coefficients.

**Table 1:** Sociodemographic and clinical characteristics of the sample

	Total (*N* = 1026)		Males (*N* = 433)		Females (*N* = 593)	
	*N*	%	*N*	%	*N*	%
Age, mean (SD) in years	*M* = 58.1	(15.5)	*M* = 63.0	(14.2)	*M* = 54.5	(15.4)
Age category						
18–39 years	154	15.0	37	8.5	117	19.7
40–49 years	121	11.8	23	5.3	98	16.5
50–59 years	220	21.4	78	18.0	142	23.9
60–69 years	269	26.2	134	30.9	135	22.8
≥ 70 years	262	25.5	161	37.2	101	17.0
Diagnosis groups						
Gastrointestinal	191	18.7	113	26.1	79	13.3
Gynecologic, breast	362	35.3	5	1.2	357	60.2
Urologic	269	26.2	235	54.3	34	5.7
Hematooncologic	126	12.3	59	13.6	67	11.3
Other	77	7.5	21	4.8	56	9.4
Time since diagnosis						
≤ 6 months	604	58.9	293	67.7	311	52.4
> 6 months	422	41.1	140	32.3	282	47.6

Confirmatory factor analyses (CFAs) were performed to evaluate the overall fit of the one-dimensional model. Fit indices were the Chi^2^ goodness-of-fit statistic, the comparative fit index (CFI), the Tucker–Lewis index (TLI), and the root mean square error of approximation (RMSEA). Hu and Bentler [38] advised that CFI and TLI values close to 0.95 are indicative of a good fit, and a RMSEA value of 0.08 or less indicates an acceptable fit of the model in relation to the degrees of freedom. CFA calculations were performed with MPlus version 6.1; all other statistics were performed with SPSS version 24.

## Results

### Sample characteristics

Of the 1053 patients who took part in the study, 27 had to be excluded because two or more of the seven ISI items had been omitted in their responses. If only one item was missing, it was replaced with the rounded mean of the remaining items. The final sample comprised 1026 patients, 433 males (42.2%) and 593 females (57.8%). Further details of the sample are given in Table 1.

The mean scores of the ISI items and the ISI sum score are presented in Table 2. All of the items contributed substantially to the sum score, the part-whole-corrected item-test-correlations ranged from 0.66 to 0.85, and Cronbach’s alpha was 0.92.

**Table 2:** Item characteristics of the ISI

No	Item	*M*	(SD)	*r*_it_	alpha del
1	Problems falling asleep	1.55	(1.21)	.67	.91
2	Problems staying asleep	2.18	(1.26)	.79	.90
3	Early awakening	1.62	(1.30)	.66	.92
4	Dissatisfaction	2.14	(1.06)	.85	.90
5	Functional impairment	1.28	(1.03)	.66	.91
6	Noticeability	1.75	(1.13)	.85	.89
7	Distress	1.86	(1.14)	.79	.90
Scale		12.39	(6.71)		alpha = .92

*M*, mean score (item range: 0–4; scale range: 0–28); *SD*, standard deviation; *r*_it_, part-whole-corrected item-test-correlation; *alpha del.*, Cronbach’s alpha if item deleted

The CFA fit indices of the one-dimensional model were as follows: Chi^2^(df) = 152.376 (14), CFI = 0.969, TLI = 0.954, RMSEA = 0.101, and SRMR = 0.026.

### Correlations with other variables

The correlations between the sleep variables and several other variables are given in Table 3. With the exception of the PHQ-9 (which also includes the PHQ-9 sleep item), all correlations of the ISI were slightly higher than those of the PHQ-9 sleep item.

**Table 3:** Correlations between the sleep scales and other variables

	ISI	PHQ-9 sleep
PHQ-9: depression	.59	.65
PHQ-2: depression	.45	.42
GAD-2: anxiety	.45	.39
ADNM-8: adjustment disorder	.57	.50
DC: fatigue	.61	.58
WAS: work ability	− .36	− .34
EORTC QoL: quality of life	− .37	− .32
ISI	-	.72

All coefficients are statistically significant with *p* < 0.001

### Sex and age differences in sleep quality

**Fig. 1:**
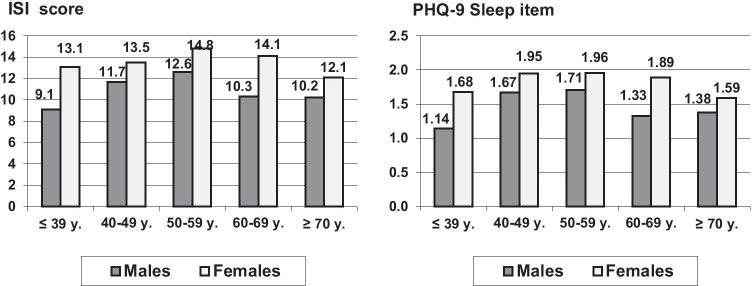
ISI and PHQ-9 sleep item mean scores, broken down by gender and age

Figures 1 shows sex and age differences in sleep problems measured with the ISI and the PHQ-9 sleep item. The overall mean scores (total sample) of the ISI and the sleep item of the PHQ-9 were *M* = 12.4 ± 6.7 and *M* = 1.65 ± 1.02, respectively. In all age groups, females reported more severe sleep problems than males did. The ISI mean scores were 13.7 ± 6.6 (females) and 10.7 ± 6.4 (males), the corresponding mean scores of the PHQ-9 sleep item were 1.83 ± 1.00 (females) and 1.41 ± 1.00 (males), resulting in sex effect sizes of *d* = 0.46 (ISI) and *d* = 0.42 (PHQ-9 sleep item). There were no linear age trends. The greatest degree of sleep problems was observed among 50 to 59-year-olds, while younger and older participants reported better sleep quality. Males and females showed roughly parallel curves, which is reflected by the very low and nonsignificant ANOVA interaction effects (see below).

The ANOVA results for testing sex and age effects on ISI sleep quality were as follows: sex: *F* = 32.4, *p* < 0.001; age group: *F* = 5.07, *p* < 0.001; sex × age group: *F* = 1.14, *p* = 0.336. The corresponding coefficients of the PHQ-9 sleep item were sex: *F* = 25.3, *p* < 0.001; age group: *F* = 5.49, *p* < 0.001; sex × age group: *F* = 1.42, *p* = 0.214.

Using the categories *no*, *subthreshold*, *moderate*, and *severe* sleep problems, the total sample was to be characterized as follows: *no* (*n* = 264; 25.7%); *subthreshold* (*n* = 362; 35.3%); *moderate* (*n* = 308; 30.0%), and *severe* (*n* = 92; 9.0%) sleep problems. For males, the percentages were as follows: *no* (32.3%), *subthreshold* (40.0%), *moderate* (23.8%), *severe* (3.9%), while for females the corresponding percentages were *no* (20.9%), *subthreshold* (31.9%), *moderate* (34.6%), and *severe* (12.6%).

### Impact of tumor type and time since diagnosis on sleep quality

Table 4 presents mean scores for the ISI and the PHQ-9 sleep item, broken down by tumor type and time since diagnosis for both sexes. While for male patients there were no significant differences between the tumor-type groups, in the females’ subsample, patients with hematooncologic cancer reported relatively few sleep problems. Time since diagnosis had no significant impact of sleep quality.

**Table 4:** Impact of clinical variables on sleep quality

	ISI				PHQ-9 sleep item			
	Males		Females		Males		Females	
	*M*	(SD)	*M*	(SD)	*M*	(SD)	*M*	(SD)
Diagnosis groups								
Gastrointestinal	11.0	(6.5)	13.9	(6.0)	1.48	(0.97)	1.90	(0.97)
Gynecologic, breast	-	-	13.5	(6.6)	-	-	1.86	(0.98)
Urologic	10.3	(6.3)	14.7	(5.9)	1.34	(0.99)	1.94	(0.95)
Hematooncologic	11.1	(6.4)	11.8	(6.9)	1.42	(1.05)	1.48	(1.08)
Other	11.9	(7.0)	15.7	(7.4)	1.71	(1.19)	1.89	(1.01)
ANOVA	*p* = 0.620		*p* = 0.017		*p* = 0.474		*p* = 0.036	
Time since diagnosis								
≤ 6 months	10.4	(6.4)	13.4	(6.6)	1.41	(0.99)	1.85	(1.00)
> 6 months	11.9	(6.4)	13.9	(6.7)	1.41	(1.02)	1.81	(0.99)
ANOVA	*p* = 0.335		*p* = 0.372		*p* = 0.990		*p* = 0.646	

ANOVA: significance of the main effect (diagnosis group or time since diagnosis) in the ANOVA with age group as covariate

### Disruptive factors

Mean scores of the items characterizing the disruptive factors are given in Table 5. The highest mean values (*M* > 1.0 on the 0–3 scale) were found for nocturnal urination, brooding, worries, and unsolved problems. Table 5 also presents the proportions of participants who responded with scores of 1 (“a bit”) or higher. Nocturnal urination and brooding were experienced by at least 75% of the patients, at least to a small degree. The highest correlations between the disruptive factors and the ISI was found for brooding (*r* = 0.58) and worrying (*r* = 0.53), while the correlation between nocturnal urination and ISI sleep quality (*r* = 0.26) was relatively low.

**Table 5:** Disruptive factors

No	Factor	*M*	(SD)	% > 0	*r* (factor, ISI)
1	Pain	0.69	(0.86)	47%	.35
2	Nocturnal urination	1.26	(0.94)	77%	.26
3	Brooding	1.25	(0.95)	75%	.58
4	Worries	1.11	(0.95)	69%	.53
5	Unsolved problems	1.02	(0.91)	67%	.46
6	Sweat	0.80	(0.95)	50%	.35
7	Hot flashes	0.75	(0.97)	56%	.35
8	Cold feeling	0.48	(0.77)	34%	.23
9	Restless legs	0.57	(0.85)	37%	.31
10	Breathing difficulties	0.23	(0.54)	18%	.19
11	Nightmares	0.46	(0.74)	34%	.36
12	Noise	0.42	(0.73)	31%	.19

*M*, mean score (range: 0–3); *SD*, standard deviation; *%* > *0*, proportion of respondents who answered at least with the category “a bit”

## Discussion

The first aim of this investigation was to test the psychometric quality of the ISI. Cronbach’s alpha (0.92) was very good. Yusufov et al. [15] compiled several studies with 12 alpha coefficients, ranging from 0.75 to 0.92. That is, the internal consistency in our study with cancer patients was at the upper end of the range. CFA results were also good. Three of the four criteria proposed by Hu and Bentler were met. We could also have calculated other CFA models. Splitting a scale into subscales generally improves the fit coefficients. Several studies found better fit indices for two- or three-factorial models [15, 16]. However, even if the number of subscales (2 or 3) was identical in the different studies, the assignment of the items to the scales was heterogeneous. We believe that adding a new factor structure based on our data would not contribute to the assessment of the quality of the ISI. Whenever ISI sum scores are calculated and compared, it is only relevant how reliable this one-dimensional scale is.

The sleep item of the PHQ-9 performed nearly as well as the ISI. The correlations between the PHQ-9 sleep item and the other scales (Table 5) were only slightly lower than the correlations of the ISI, and the age and gender effects in the ANOVAs were of nearly equal magnitude for the PHQ-9 sleep item and the ISI sum score. In addition, the sex and age effects and the differences in the tumor type showed similar patterns for the PHQ-9 sleep item and the ISI. If a study already includes the PHQ-9 for measuring depression, the PHQ-9 sleep item seems to be a roughly sufficient surrogate for a longer questionnaire such as the ISI. Unfortunately, our study did not include a gold standard which could be used to compare the sensitivity and specificity coefficients of both sleep instruments. In a study with Iranian cancer patients [2], the correlations between the ISI scores and the scores of two other sleep questionnaires, the Athens Insomnia Scale and the Pittsburgh Sleep Quality Index, were 0.64 and 0.58, respectively. In our study, the correlation between the ISI and the sleep item of the PHQ-9 was even somewhat higher (*r* = 0.72).

The magnitude of sleep problems in our sample was high. There are no normative studies for the ISI, but comparisons between our cancer patients and the general population can be performed using the PHQ-9 sleep item. The mean score obtained in the general population of the PHQ-9 sleep item was *M* = 0.63 (SD = 0.75) [39], which is much lower than the score in our sample (*M* = 1.65, SD = 1.02). This represents a large difference (effect size *d* = 1.15) and underlines the importance of sleep disturbances in patients with cancer.

ISI mean scores in samples which exclusively comprised patients with sleep disorders are even higher than the mean (12.4) of our cancer patients’ sample, e.g., 16.4 in a Korean study [40] and 16.9 in an Italian study [17]. A study with German cancer patients reported a lower ISI mean score of 8.7 [3]. Surveys with non-clinical populations also yielded lower mean scores: 7.0 (employees of the police force and emergency response service corps) [41], 6.6 (students) [41], 6.7 (adolescents) [41], and 9.8 (general population aged 65 and above) [42]. Dieck et al. [43] investigated two groups, (a) people from the general population who were recruited to attend a sleep training group (ISI mean = 16.8) and (b) a convenience sample of people who were offered the opportunity to have their sleep quality tested (ISI mean = 9.8). All these samples of non-patients have certain peculiarities and are not representative of the general population. Normative studies of the ISI would be very helpful for assessing sleep problems among cancer patients.

Females reported having markedly more sleep problems than males in our study. This sex effect is a general phenomenon. Normative studies using different questionnaires confirm this sex difference [19, 20]. However, the age trend was interesting in our sample. In the general population, Tibubos et al. [19] found an increase in sleep problems with increasing age, while Hinz et al. [20] did not. In our sample of cancer patients, we observed a non-linear trend with the most severe sleep problems occurring among 50- to 59-year-olds. Because people of this age are generally still participating in the work force, being ill with cancer impacts not only their health but their professional existence as well. Older people might consider their situation a little bit more relaxed since the occupational problems might disappear for the retired people or for those shortly before retirement. It has repeatedly been shown that sleep problems among unemployed/retired people are much higher than those who work full-time or half-time [19].

Several studies have analyzed the impact of sociodemographic and clinical factors, e.g., tumor type [16] or pain intensity [42], on sleep quality, but these studies did not investigate the specific disruptive factors to which the participants attribute their sleep problems. In our study, we investigated 12 of these factors. The main result was that, of the factors we included, mental factors played a greater role than physical factors. The highest correlations with sleep quality (*r* > 0.50) were found for brooding and worries, while the association between sleep problems and pain, sweat, and restless legs were markedly lower.

According to Spielman and Glovinsky [44, 45], there are three categories of factors involved in the development of sleep problems: predisposing factors, precipitating factors, and perpetuating factors. The disruptive factors included in our study mostly belong to the second category of precipitating factors; however, a clear assignment is not always possible. The most relevant factors of mental distress may follow from personality traits (category 1), or they may be caused by disease and treatment (category 2). The factors that proved to be most relevant in our study (brooding, worries) in terms of correlations with sleep problems may be caused by the disease (precipitating factors) or by personality traits (predisposing factors).

The comparison of the factors analyzed in this study shows that the mental factors are at least as relevant as the physical ones. Helping patients brood and worry less might be more effective in solving sleep problems than only reducing physical symptoms. Treatment programs for regaining good sleep should consider the importance of the role mental factors play. Cognitive-behavioral therapy for insomnia (CBT-I) has demonstrated its effectiveness in cancer patients and cancer survivors [46, 47].

Some *limitations* of the study should be mentioned. We assessed the degree of patient’ subjectively experienced sleep problems and did not examine objective criteria of sleep quality or diagnoses of insomnia based on semi-structured interviews. Subjective and objective measurement methods can differ substantially [48, 49]. The sample of cancer patients who were all receiving treatment in a rehabilitation clinic is not totally representative of cancer patients in general. Those cancer patients who do not perceive severe physical or mental problems might be underrepresented in the rehabilitation clinic. However, a study which compared the QoL of hospitalized cancer inpatients, patients in treatment at a rehabilitation clinic, and cancer outpatients did not detect significant differences between those groups [50]. The PHQ-9 is only one of several questionnaires which contain a single sleep item; we cannot generalize that single-item scales are generally roughly as effective in detecting sleep problems as the seven-item ISI. In our study, we analyzed several disruptive factors, which, however, do not belong to a validated questionnaire, and the reliability and validity of these variables is unknown. In addition, even statistically significant associations between these factors and the ISI scores cannot be interpreted in a causal way: worries may result in sleep problems and vice versa.

## Conclusion

The results of the study underline the importance of sleep problems experienced by cancer patients. The ISI proved to be a reliable instrument for measuring the subjectively perceived sleep quality, the instrument can be recommended for clinicians and researchers. Though the sleep item of the PHQ-9 also performed relatively well in detecting sleep problems, we do not recommend to use such a single-item measure in clinical routine instead of a validated questionnaire such as the ISI. This study also included disruptive factors and found that mental factors played a greater role than physical factors for explaining sleep disturbances.

In conclusion, the ISI proved to be a reliable instrument for measuring sleep quality in cancer patients. Comparisons between groups with different types of cancer should always take into account that sex differences might contribute to the differences in the results. Normative studies with the ISI, derived from representatively selected people of the general population, would be very useful for interpreting the results of the patients’ responses.

## Data Availability

The data that support the findings of this study are available from the corresponding author upon reasonable request.

## Code availability

We used the statistics software SPSS version 24 and MPlus version 6.1.
